# Impact of Food-Derived Bioactive Compounds on Intestinal Immunity

**DOI:** 10.3390/biom11121901

**Published:** 2021-12-18

**Authors:** Christian Zimmermann, Anika E. Wagner

**Affiliations:** Institute of Nutritional Sciences, Justus-Liebig University, Wilhelmstrasse 20, 35392 Giessen, Germany; Christian.A.Zimmermann@ernaehrung.uni-giessen.de

**Keywords:** immune system, mucosal immunity, plant bioactives, prebiotics, probiotics

## Abstract

The gastrointestinal system is responsible for the digestion and the absorption of nutrients. At the same time, it is essentially involved in the maintenance of immune homeostasis. The strongest antigen contact in an organism takes place in the digestive system showing the importance of a host to develop mechanisms allowing to discriminate between harmful and harmless antigens. An efficient intestinal barrier and the presence of a large and complex part of the immune system in the gut support the host to implement this task. The continuous ingestion of harmless antigens via the diet requires an efficient immune response to reliably identify them as safe. However, in some cases the immune system accidentally identifies harmless antigens as dangerous leading to various diseases such as celiac disease, inflammatory bowel diseases and allergies. It has been shown that the intestinal immune function can be affected by bioactive compounds derived from the diet. The present review provides an overview on the mucosal immune reactions in the gut and how bioactive food ingredients including secondary plant metabolites and probiotics mediate its health promoting effects with regard to the intestinal immune homeostasis.

## 1. Introduction

The gastrointestinal tract resembles the most substantial part of the digestive system, being responsible for the digestion and absorption of nutrients and excretion of metabolic products. In addition, it comprises the largest and most complex part of the immune system with the strongest antigen contact within an organism. Therefore, it is essential that it can discriminate between harmful and harmless antigens like food ingredients and commensal microorganisms. On the one hand, if pathogens overcome the mucosal barrier, effective immune responses have to be initiated while on the other hand hypersensitive immune reactions to non-pathogenic microorganisms need to be prevented [1].

To get in contact with immune cells, antigens have to cross the physical barriers including the residing commensal microorganisms, the mucus layer and eventually the epithelial barrier. The mucus which separates into a sterile inner and a permeable colonized outer layer is secreted by goblet cells that shape together with Paneth cells, enteroendocrine cells and enterocytes the epithelial barrier of the small intestine. The permeability of the epithelial barrier is controlled by intercellular junctions including occuldin and claudins. The intestinal barrier is further maintained by antimicrobial peptides (AMPs) like defensins that are either constitutively produced by or generated through an interaction of bacterial structures such as lipopolysaccharides (LPS) with epithelial cells which in consequence counteract invading pathogens [2].

Luminal antigens are able to translocate via M-cells to the gut-associated lymphoid tissue (GALT) including Peyer’s patches and isolated lymphoid follicles. Dendritic cells (DCs) with transepithelial dendrites are also able to recognize luminal antigens. Soluble antigens, depending on type and size of the antigen, are also able to diffuse through epithelial tight junctions or may be transferred through transepithelial routes [1,3].

Once antigens have passed the epithelial barrier they can induce both, the innate and the adaptive immune response. The innate immune response represents the first line of the immunological defense against pathogens and acts rather unspecific. The conserved structures of bacterial pathogens known as pathogen associated molecular patterns (PAMPs) can be recognized by specific cells including DCs, macrophages, intestinal epithelial cells and myofibroblasts through pattern recognition receptors (PRRs) which in consequence induce inflammatory responses against invading pathogenic bacteria [2].

In contrast to the innate immune system, the adaptive immune system is highly specific and adaptable for antigens. Once antigens pass the epithelial barrier they can be processed by professional antigen presenting cells (APCs) such as DCs. Besides antigen-loaded APCs, unbound antigens can also translocate to T-cell areas, B-cell follicles or mesenteric lymph nodes where they are processed and presented by APCs and subsequently interact with naïve T-cells or B-cells, respectively, to induce the adaptive immune response. Lamina-propria-derived APCs and even enterocytes are able to present antigens to local T-cells that either differentiate into a) Th0 cells that may further differentiate into Th1 cells to eliminate intracellular pathogens, or into b) Th2 cells to defend against invading parasites, or into c) Th17 cells that are involved in the clearance of extracellular pathogens.

An effective immune response against pathogens depends on different components of the immune system acting both, individually and cooperatively [1,2,3]. However, it has to be considered that exaggerated immune reactions to harmless antigens have to be prevented indicating the need of a tightly regulated intestinal immune system. In this regard, regulatory T-cells (T_reg_) play a central role in maintaining immune homeostasis and immunological tolerance as they suppress abnormal immune reactions against e.g., dietary antigens and the commensal microbiota [2,4]. Once this homeostatic state is disrupted it may result in inflammatory intestinal disorders including inflammatory bowel disease or celiac disease being characterized by an increased infiltration of luminal antigens [1,3].

The intake of specific nutrients and bioactive compounds may target the intestinal immune system and the intestinal microbiota consequently modulating the course of certain diseases. One type of bioactive compounds are secondary plant metabolites that are present in plants and consequently in fruits and vegetables. They are produced by the plant itself in order to defend itself against exogenous insults e.g., UV radiation, predators and pathogenic microorganisms [5]. These secondary metabolites are associated with various health promoting properties in different organisms, including invertebrates such as *Drosophila melanogaster*, laboratory rodents, and humans [6,7].

Another possibility to modulate the intestinal microbiota is the ingestion of specific microorganisms that are able to survive the gastrointestinal passage. These so-called probiotic microorganisms have been associated with health-promoting effects in relation to the intestinal immune system and have been discussed as possible agents in the prevention and/or the therapy of several inflammatory diseases [8,9]. In addition, prebiotic compounds have been shown to modulate the composition of the intestinal microbiota which may support probiotics in mediating their potential health-promoting properties. With regard to prebiotics, it has to be taken into account that these compounds mediate effects that go beyond the influence on the microbiota, specifically direct effects on the intestinal immune system [10,11].

This review aims to provide an overview of the immune responses of the intestinal mucosa and how bioactive food ingredients such as secondary plant metabolites and pre- and probiotics can potentially influence the gut immune homeostasis to mediate their health-promoting effects.

## 2. Effect of Food-Derived Bioactive Compounds on the Gastrointestinal (Mucosal) Immune System

The following sections introduce various nutrients and plant bioactives and explain how they interact with factors and/or signaling pathways associated with the immune system.

### 2.1. Flavonoids

Flavonoids represent a class of polyphenols that are associated with various health-promoting properties including anti-inflammatory and anti-oxidative effects [12]. Anthocyanins (AC), a subgroup of the flavonoids, are phytochemicals that are present in relatively high amounts in numerous fruits and vegetables where they induce different colors and protect the plants from various predators [13]. These phytochemicals also affect the gastrointestinal tract which may be one reason for the systemic effects being observed following the consumption of dietary plant bioactives [14].

#### Anthocyanins

AC have been demonstrated to reverse adverse effects of a high fat diet (HFD) including an impaired gut barrier. In mice on a HFD AC have been shown to improve the integrity of the gastrointestinal barrier [15] which is supported by results from AC-supplemented HFD-exposed mice that express higher levels of ileal tight junction proteins such as occludin and claudin-1 and mucin 2 (MUC2), a major protein of the mucus layer representing the first line of immunological defense in the gut [16]. Moreover, the HFD-induced adverse effects on the intestinal microbiota of these mice which was reflected in a higher *Firmicutes*/*Bacteroidetes* ratio as well as a lower abundance of *Akkermansia* has been reversed by AC supplementation [15]. *Akkermansia* abundance has been supposed to play an important role in the maintenance of the gut homeostasis as patients suffering from inflammatory bowel diseases and metabolic disorders, both diseases associated with a destruction of gut homeostasis, host lower numbers of these bacteria in their intestines [17].

AC supplementation has also been demonstrated to exhibit potent anti-inflammatory activities in the gut. In co-culture cell systems mirroring the situation of the intestinal epithelium, AC from purple carrots and potatoes [18] as well as the anthocyanidin cyanidin-3 glucoside (C3G) [19] mediated anti-inflammatory effects by targeting the NFκB pathway consequently resulting in lower levels of pro-inflammatory cytokines. These results have also been confirmed in mice where a treatment with C3G mediated anti-inflammatory effects resulted in a decrease of pro-inflammatory cytokines and an increase of the chemokine CCL-22 in the colonic tissue as well as in the mesenteric lymph nodes [20]. Interestingly, a high expression of CCL-22 has been associated with increased numbers of T_reg_ cells which are central players in immune homeostasis and specifically in mediating immune tolerance [21]. AC have also been associated with anti-carcinogenic effects. By feeding AC-rich freeze-dried cloudberries to Apc^Min^ (multiple intestinal neoplasia/+) mice the numbers and sizes of their adenomas have decreased [22] which may be the consequence of the impaired intestinal inflammation being reflected in a lower ratio of intraepithelial to all mucosal CD3+ T lymphocytes [23].

### 2.2. Phenolic Acids

Phenolic acids are plant bioactives that can be divided into two major groups—the hydroxy-benzoic acids with vanillic acid and gallic acid as representatives and the hydroxycinnamic acids with ferulic acid and curcumin as representatives [24].

#### 2.2.1. Hydroxybenzoic Acids

The hydroxy-benzoic acids vanillic acid and gallic acid have been demonstrated to exhibit anti-inflammatory effects in cell culture and laboratory rodents. In murine LPS-activated peritoneal macrophages vanillic acid has significantly lowered the levels of pro-inflammatory markers such as tumor necrosis factor α (TNFα) and IL-6 as well as of cycloxygenase 2 (COX-2), prostaglandin E2 (PGE2) and nitric oxide [25]. A treatment with gallic acid has caused a significant decrease of the inflammation-associated NFκB pathway in LPS-activated murine RAW264.7 macrophages [26]. This has been confirmed in mice with a dextran sodium sulfate (DSS)-induced colitis where gallic acid significantly inhibited the colonic inflammation by affecting NFᴋB and interleukin 6 (IL-6)/pSTAT3Y705 activation [27]. In a murine allograft model a treatment with gallic acid has increased the T_reg_ cell population causing a decrease of T-cell activation and lower T-cell numbers suggesting a potential use of this compound in diseases with an excessive activation of the immune system including e.g., autoimmune diseases [28].

#### 2.2.2. Hydroxycinnamic Acids

In LPS-exposed THP1 macrophages a treatment with ferulic acid resulted in a significant down-regulation of IL-1β and IL-6 levels [29]. Systemic inflammatory biomarkers such as IL-1β, IL-4 and IL-6, have also been significantly improved in rats receiving a ferulic acid-supplemented HFD [30]. Curcumin represents a bis-α,β-unsaturated β-diketone of two ferulic acid units [31] and has been reported to exhibit immune-modulating properties through an interaction with immune cells [32]. Curcumin-exposed DCs from murine bone marrow derived dendritic cells (BMDC) have induced the differentiation of naïve CD4+ T-cells into intestine protective T_reg_ [33]. In a co-culture Caco-2 cell model a pre-treatment with curcumin also significantly improved the intestinal barrier by reversing the leptin-induced barrier dysfunction through e.g., an up-regulation of gene expression levels of various tight junction proteins and a decrease in gene expression of pro-inflammatory cytokines such as TNFα and IL-6 [34]. Mice lacking IL-10 (IL-10−/− mice) spontaneously develop a Th1-driven colitis. This colonic inflammation especially with regard to the colon morphology has only moderately improved following a treatment with curcumin. In colonic explants and mesenteric lymph node cells of these IL-10−/− mice, IL-12/23p40 and interferon γ (IFNγ) secretion increased which has been, however, not reversed following curcumin supplementation. These results suggest that curcumin mediates its health-promoting properties only in the presence of IL-10 [35]. McFadden and co-workers [36] have also performed experiments with IL-10−/− mice and—similar to the results of Larmonier and colleagues [35]—did not observe any effects of curcumin-treatment on the mucosal immune response while in azoxymethane (AOM) treated IL-10−/− mice curcumin exhibited a limited effect on tumorigenesis. Interestingly, in curcumin-exposed AOM/IL-10−/− mice effects on the intestinal microbiota have been observed which has been reflected in a higher bacterial richness, an increased abundance of *Lactobacillales* and a lower abundance of *Coriobacterales* consequently leading to a more diverse colonic microbiota [36]. Curcumin has been suggested to mediate its anti-inflammatory effect also by affecting the innate immune response. This has been demonstrated in mice where a curcumin treatment on the one hand decreased the recruitment of neutrophils and on the other hand mediated direct effects on the polarisation of neutrophils, chemotaxis and chemokinesis [37]. In the context of inflammatory bowel disease, the transepithelial neutrophil migration results in an impaired barrier function, sustained inflammation and tissue damage which were inhibited by curcumin potentially through affecting chemokine expression, chemotaxis and chemokinesis [38].

### 2.3. Stilbens

3,5,4′-trihydroxy-trans-stilbene, also known as resveratrol, is a non-flavonoid polyphenol that is classified into the group of stilbens. It can be found in high amounts in the skin of grapes and in red wine and has therefore been suggested to be a main player in the so-called French Paradox [39]. Resveratrol has been further shown to affect the intestinal immune function [40]. It has been verified by Mayangsari & Suzuki [41] in the human intestinal cell line Caco-2 that a pre-treatment with resveratrol resulted in a significant down-regulation of both the NFκB-signaling pathway and an inhibition of the kinases ERK and JNK. Similar effects have been observed by Panaro and colleagues [42] in Caco-2 and SW480 cells in which resveratrol significantly lowered the LPS-mediated pro-inflammatory responses which have been suggested to be mediated through a down-regulation of the NFκB pathway as well as a decreased expression of toll like receptor (TLR) 4 and the inducible NO synthase (iNOS). The latter contributes to the decreased production of NO which has been connected with cellular injury and impaired barrier function in the gut [43]. There are also contradictory results regarding the anti-inflammatory potential of resveratrol. For instance, Romier and colleagues [44] have not observed any protective effects of resveratrol in Caco-2 cells. In a reporter gene assay, IL1β, TNFα and LPS significantly induced the NFκB-dependent luciferase activity which has, however, not been lowered by resveratrol treatment. This has also been true for the IL-1β—induced IL-8 secretion in these cells, which further increased by resveratrol treatment [44]. However, most of the studies available dealing with the effects of resveratrol on intestinal inflammation document anti-inflammatory effects on the molecular as well as on the microscopic and tissue level.

In murine models of acute [45] and chronic colitis [46] an oral treatment with resveratrol has improved inflammatory markers such as pro-inflammatory cytokines and serum amyloid A (SAA) as well as tight junction proteins. During acute colitis, the percentage of CD4+ T-cells in the mesenteric lymph nodes of resveratrol-treated mice have remained at normal levels while CD4+ T-cell numbers in the lamina propria decreased. The proportion of macrophages in mesenteric lymph nodes and the lamina propria were also significantly lower compared to the DSS-exposed control animals. DSS-treatment has significantly decreased the histone-deacetylase sirtuin 1 (SIRT1) in lamina-propria-derived lymphocytes being counteracted by the treatment with resveratrol pointing towards an involvement of epigenetic pathways [45]. In a therapeutic approach Yao et al. [47] have exposed mice to DSS for 7 days to induce a colitis. Following, mice have been either subjected to an oral treatment with resveratrol or with vehicle for further 7 days. Resveratrol has affected the levels of cytokines including IL-10, TGF1β, IL-6 and IL-17 in both, plasma and the colonic tissue. Low resveratrol concentrations have regulated the T_reg_/Th17 balance by a reduction of Th17 cells, while high concentrations of resveratrol resulted in a decrease of Th17 cells and a simultaneous increase of T_reg_ cells. Similar results have been observed by Sánchez-Fidalgo and colleagues [48] in a preventive approach in which mice have been either subjected to a standard diet or a resveratrol-supplemented diet for 30 days. Subsequently, a chronic colitis has been induced lasting for another 21 days. Dietary resveratrol has significantly improved colitis symptoms which has been reflected in a lower disease activity index compared to control animals as well as a decrease of pro-inflammatory and an increase of anti-inflammatory cytokines in the murine colonic tissue. The authors have also reported that all resveratrol-fed mice survived until the end of the study while DSS-treated mice on the control diet exhibited a mortality rate of 40%. Furthermore, inflammation-associated enzymes such as prostaglandin E (PGE) synthase-1, COX-2 and iNOS were significantly down-regulated by p38, a MAPK signaling pathway. These results have been confirmed by Larrosa and co-workers in laboratory rats [49]. One mg resveratrol/kg body weight/day—a dose that can be achieved by dietary intake in humans—has been applied to rats for 25 days followed by a colitis-induction through DSS-exposure during the last 5 days of the experiment. Besides observing a significant decrease of inflammatory markers such as PGE2 and COX-2, a significant increase in the number of health promoting gut bacteria (*Lactobacillus* and *Bifidobaceria*) has been detected in feces of rats treated with resveratrol [49]. Resveratrol has significantly increased the number of myeloid-derived suppressor cells (MDSC) in the lamina propria and the spleen of colitic IL-10−/− mice [50]. MDSC have been shown to proliferate during intestinal inflammation in a mouse model of colitis mediating immune suppression and consequently affecting immune regulation [51].

Resveratrol treatment has significantly reduced the symptoms of colitis in IL-10−/− mice such as weight loss and the serum level of the acute phase protein SAA, a well-documented biomarker for colitis severity [50]. Under chronic colitis the immunglobulins IgG and IgA are secreted from the intestinal mucosa into the lumen resulting in high fecal levels of IgG and IgA. A treatment with resveratrol has counteracted these inflammation-associated mucosal responses and restored normal fecal IgG and IgA levels in IL-10−/− mice [50]. The protective effects of resveratrol have also been reflected by an increased expression of tight junction proteins and a decreased neutrophil infiltration in the colon consequently contributing to a better intestinal barrier function in colitic mice [41]. Interestingly, in a randomized controlled trial a daily dose of 500 mg resveratrol administered over a period of 6 weeks to patients with active ulcerative colitis (UC) has significantly lowered inflammatory biomarkers in plasma (TNFα, high sensitivity-C-reactive protein) as well as NFκB activity in patient-derived PBMCs. In addition to a significant reduction in the clinical colitis activity index also the quality of life index significantly improved in the UC patients by resveratrol treatment while the parameters measured remained unchanged in the placebo group [52].

### 2.4. Glucosinolates

Glucosinolates are secondary plant metabolites present in plants of the family *Brassicaceae*. Several health-promoting properties have been attributed to these compounds, such as anti-inflammatory and anti-carcinogenic effects. Epidemiological studies point towards an association between a high consumption of cruciferous vegetables and a lower incidence for different types of cancer including colorectal cancer [53,54]. As it has been suggested that a chronic inflammation of the intestine predisposes for the development of colorectal carcinoma [55], the glucosinolate’s protective effects may be ascribed to its anti-inflammatory potential, especially in the gastrointestinal tract. The observed health-promoting effects are, however, not attributed to the glucosinolates itself but to their corresponding breakdown products [56,57]. Depending on the reaction conditions, such as temperature and pH, different products emerge: nitriles, thiocyanates and isothiocyanates. The glucosinolates are present in the intact plant while upon disruption by cutting or chewing—they get in contact with myrosinase, an enzyme located in another plant organell, which under neutral pH causes the release of isothiocyanates [58,59]. Another possibility of isothiocyanate release from glucosinolates after ingestion of Brassica-containing foods is through myrosinase-expressing bacterial species of the intestinal microbiota [60].

Studies investigating effects of glucosinolates on the intestinal immune system are limited, while some publications are available looking into the immune-modulating properties of isothiocyanates. The intraperitoneal administration over a period of 5 days of sulforaphane (SFN), an isothiocyanate generated from its precursor glucoraphanin which can be found in high amounts in Broccoli, has significantly enhanced the numbers of white blood cells in BALB/c mice and significantly increased the antibody titre following immunization with sheep-derived red blood cells. Exposing mice to SFN prior to an induction of systemic inflammation by LPS, has resulted in significantly lower levels of the pro-inflammatory cytokines TNFα, IL-1β and IL-6 in their plasma [61]. The anti-inflammatory potential of SFN has also been confirmed in a murine DSS-colitis model in which the SFN pre-treatment significantly lowered symptoms of intestinal inflammation, including weight loss, pro-inflammatory markers, infiltration of monocytes and the pro-inflammatory microRNA-155 [62]. In mice with an LPS-induced systemic inflammation SFN has significantly attenuated the gene expression of pro-inflammatory cytokines in the intestine suggesting an impact on the homeostasis of the intestinal barrier [63]. SFN may also mediate its anti-inflammatory effects by counteracting the inflammation-induced dysbiosis of the gut microbiota. Zhang and colleagues [64] have detected an improvement of typical indicators of inflammation in a DSS-induced colitis model by SFN pre-treatment which has been accompanied by changes in the composition of the intestinal microbiota. DSS-treated mice exhibited higher levels of *Firmicutes* and lower ones of *Bacteroidetes* and in consequence a higher *Firmicutes*/*Bacteroidetes* ratio which has been associated with negative health outcomes including obesity and inflammatory bowel syndrome [65]. Besides SFN other brassica-derived compounds have been investigated for their anti-inflammatory potential in the context of gut inflammation. Allyl-isothiocyanate (AITC)—the hydrolyzation product of its precursor sinigrin, which is present in mustard—has significantly inhibited symptoms of intestinal inflammation in a mouse model of acute colitis which was also reflected on the cellular level where AITC decreased the infiltration of macrophages and other immune cells [66,67]. AITC treatment has also counteracted the DSS-induced loss of goblet cells in the colonic tissue of colitic mice which has been accompanied by a less pronounced depletion of mucin and epithelial cells on the surface [67].

Several studies have investigated the immune-modulating properties of isothiocyanates and other break-down products of glucosinolates on the immune system in cell-based systems. In the enterocytic cell lines Caco-2, HT-29 and SW480 SFN-treatment has resulted in an increase of the antimicrobial peptide human β-defensin-2 (HBD-2) on both, the mRNA and the protein level. HBD-2 exhibited antimicrobial effects by a permeabilization of bacterial membranes [68] and has additionally mediated chemoattraction to immune cells [69]. The immune-modulating effects have also been observed in LPS-induced murine macrophages, where SFN and AITC exhibited significant dose-dependent anti-inflammatory properties, this being reflected by significantly lower levels of inflammatory markers [70]. One of these inflammatory markers was microRNA-155 indicating a potential involvement of epigenetic mechanisms. This is supported by the fact that the structurally similar isothiocyanate SFN is a well known histone deacetylase (HDAC) inhibitor [71]. Rajendran et al. [72] and Qu and colleagues [73] have also suggested HDAC inhibition by AITC as a modulator of the innate immune response in cultured intestinal cells and DCs. The anti-inflammatory potential of AITC has also been confirmed by Kim and co-workers in murine monocytes in which AITC-treatment caused lower protein levels of the phosphorylated NFκB p65 subunit as well as decreased mRNA levels of pro-inflammatory cytokines [67]. The authors have also analyzed the expression of tight junction proteins that are essentially involved in the intestinal epithelial barrier. In Caco-2 cells a treatment with DSS has decreased the expression levels of the tight junction protein zonula occludens 1 (ZO-1) which were dose-dependently counteracted by AITC. At the same time, DSS-mediated MUC-2 depletion in LS174T-cells that are used as a model system for the mucus-producing goblet cells, were significantly induced by exposing these cells to increasing concentrations of AITC [67]. A SFN-treatment of classically activated human monocytes from the THP1 cell line has resulted in anti-inflammatory effects reflected in significantly lower mRNA levels of pro-inflammatory cytokines [74]. In contrast to the well-documented anti-inflammatory potential of glucosinolates and their break-down products, Liu and co-workers [75] have observed an increase of inflammatory processes in human Jurkat T-cells. However, this has been shown only for pharmacological but not for lower doses of the glucosinolate-derived break-down-products di-indolylmethane (DIM) and indole-3-carbinol (I3C).

**Figure 1:**
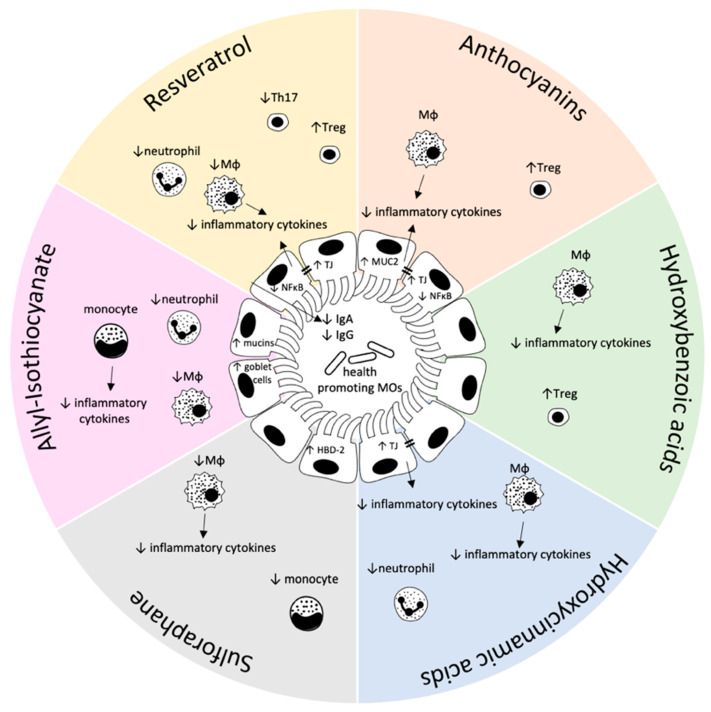
Proposed effects of plant bioactives on the gastrointestinal immune system. Detailed information on the modes of action are provided in the main text. Mɸ: macrophage, TJ: tight junction, MOs: microorganisms, MUC2: mucin 2, T_reg_: regulatory T-cell, Ig: immune globulin, Th17: T helper 17 cell, HBD-2: human β-defensin-2.

Table 1 provides an overview on the effects of these compounds on inflammatory markers in different model systems. Figure 1 summarizes the proposed effects of food-derived plant bioactive compounds on the intestinal mucosal immune system.

### 2.5. Probiotics and Prebiotics

A change in the intestinal microbiota known as dysbiosis has been associated with various inflammatory diseases, e.g., inflammatory bowel disease and diabetes type II [76]. Although the underlying mechanisms are currently not fully understood, it has been documented that certain diseases could be improved or even prevented by modulating the composition of the microbiota [77]. Therefore, a modulation of the intestinal microbiota by the application of probiotics and/or prebiotics could be an effective strategy in the treatment of inflammatory disorders.

#### 2.5.1. Probiotics

**Figure 2:**
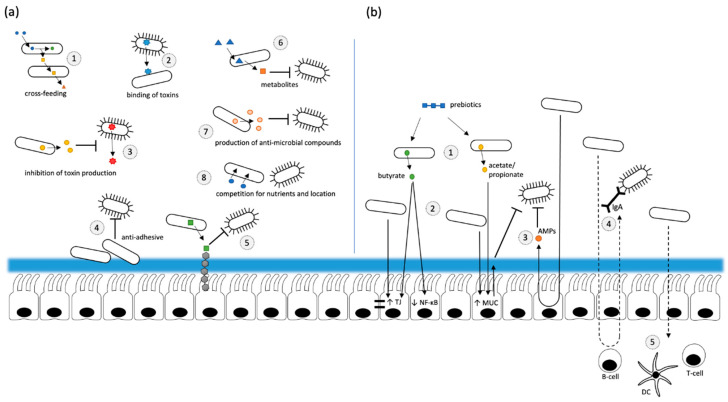
(**a**) Indirect probiotic effects on the intestinal barrier. By cross-feeding, generated metabolites from one bacterial strain may be utilized by another strain resulting in the generation of various other metabolites consequently affecting the gut barrier (1). The damaging effect of pathogen-generated toxins may be attenuated through toxin binding (2) and an inhibition of toxin production (3). A direct interaction of pathogenic bacteria with the host’s intestinal epithelial cells may be prevented by both, a formation of biofilms (4) and a competition for binding sites (5). The growth of intestinal pathogens may be inhibited by different mechanisms mediated by probiotics: Either through specific probiotic metabolites (6), the production of anti-microbial compounds (7) or a competition for nutrients/localization (8). (**b**) Direct effects of probiotics on the host. The intestinal barrier may be strengthened by the generation of short chain fatty acids such as butyrate, propionate and acetate through a metabolization of prebiotics (1) as well as through direct interactions of probiotics with epithelial cells (2) consequently improving tight junctions (TJ) and mucin (MUC) production and inducing anti-inflammatory effects. A potential contact of pathogens with the intestinal barrier may be inhibited by the generation of antimicrobial peptides (AMPs) (3) and the secretion of IgA (4). Additionally, probiotics may directly affect different intestinal immune cells (e.g., dendritic cells (DC), or T-cells) and consequently the intestinal inflammatory response (5).

Probiotics are defined as “live microorganisms that, when administered in adequate amounts, confer a health benefit on the host” [9]. Therefore, different microbial strains may be considered for a potential treatment such as different genera of lactic acid bacteria, including *Lactobacilli, Streptococci, Pediococcus, Enterococcus* or *Bifidobacteria* [78,79] but also yeasts such as *Saccharomyces* *boulardii* or *Saccharomyces cerevisiae* [80]. The present knowledge of the underlying health-promoting mechanisms of these probiotics have been mainly obtained from in vitro studies or animal models. They involve a) direct effects of probiotics on other microorganisms present and b) effects resulting from an interaction of the probiotic bacteria with the host (Figure 2) [81]. Particularly, cross-feeding, a competition for nutrients and its niche, anti-adhesive and anti-invasive effects, production of antimicrobial substances and organic acids as well as antitoxin effects have been demonstrated for probiotics to mediate their effects on other microorganisms [81,82]. Cross-feeding has been considered as an important factor for the intestinal production of butyrate, a short chain fatty acid (SCFA) known to exhibit beneficial effects on the intestinal barrier, energy metabolism and homeostasis [83,84]. For example, it has been shown in vitro that the metabolization of glycans by *Bifidobacteria* leads to the formation of acetate and lactate, both SCFAs that can be used by other bacteria, e. g. *Faecalibacterium prausnitzii*, as substrates to produce butyrate [85,86,87]. In addition to its beneficial effects on the intestinal energy metabolism, the intestinal barrier function and the modulation of the immune response, butyrate has been controversially discussed with regard to its role in obesity [88].

Each bacterial species shows different hierarchical preferences with regard to the utilization of substrates. As substrate restriction represents an important factor for the intestinal colonization, the use of specific niches for nutrient utilization may be of major importance to induce the permanent colonization with a specific bacterial strain [89,90,91]. However, it has to be considered that the colonization of probiotic strains in the gut is mostly transient and high survival rates of these strains are only detectable a few days after ingestion [92,93,94]. Maldonado-Gomez et al. [95] have shown that a daily oral administration of *Bifidobacterium longum* AH1206 over a period of 14 days persisted in 30% of the included subjects for 6 months depending on phylogenetic limitations and the availability of resources.

Pathogenic bacteria attaching to the mucosa directly interact and consequently damage the tissue, which may be inhibited by a treatment with probiotics [96]. Anti-adhesive effects of probiotics on pathogenic bacteria have also been suggested as a protective mechanism resulting either from a competition for the same receptor or from an induction of mucin production. The latter has been demonstrated for *Escherichia coli Nissle* 1917 as well as for *Lactobacillus plantarum* 299v and *Lactobacillus rhamnosus* GG in the intestinal cell line HT-29 where these probiotic bacterial strains induced the gene and protein expression levels of MUC2, MUC5 and/or MUC3 [97,98]. Besides an influence on mucin production, the degradation of pathogen-binding receptors, the production of receptor analogues and the formation of biofilms have also been discussed as potential mechanisms to prevent bacterial adhesion [81]. A protein named binding-inhibitory factor (BIF) produced by *Bifidobacterium longum* BL1928 affected the interaction of enterotoxigenic *Escherichia coli* Pb176 with human HCT-1 epithelial cells, presumably by inhibiting the binding of the pathogen to the glycolipid binding receptor gangliotetraosylceramide [99]. In a colitis mouse model *Saccharomyces boulardii* attenuated the adherence of *Citrobacter rodentium* to intestinal cells which was potentially mediated by effects on the pathogen’s virulence factors such as the type III secretion system and in consequence ameliorated gut inflammation [100]. However, anti-adhesive effects are mainly dependent on the probiotic bacteria involved and are therefore very specific. Interestingly, in an in vitro adhesion assay, bacteria classified as probiotics have also caused an increase in adhesion of some pathogenic bacteria to the intestinal mucus, although to a minor extent [101]. Probiotic bacteria have also been shown to inhibit the invasion of pathogenic bacteria to epithelial cells, consequently preventing them from infection. For example, it has been shown in CCL-6 and Caco-2 epithelial cells that the probiotic bacteria *Lactobacillus casei* DN-114 001 successfully inhibited the invasion of adherent-invasive *Escherichia coli* isolated from patients with Crohn’s disease [102]. Another anti-invasive mechanism mediated by probiotic bacteria may result from their production of antimicrobial substances which may compete with pathogenic bacteria for binding sites. Various strains of *Lactobacilli* have been able to produce organic acids, bacteriocins or certain antibiotics that in consequence impair the effect on pathogenic bacteria including *Streptococcus agalactiae* and *Listeria monocytogenes* [103,104]. For several strains of *Bifidobacterium* the production of antibacterial compounds is described. The bacteriocin bifidocin B produced by *Bifidobacterium bifidum* NCFB 1453 has been reported to exhibit inhibitory activity against several pathogenic bacteria in vitro including *Listeria monocytogenes* [105,106]. Strain UCC118 of *Lactobacillus salivarius* has been shown to produce the bacteriocin Abp118 which protected mice from infections with *Listeria monocytogenes* [107]. The inhibition of the growth of pathogenic bacteria has also been documented for SCFA, which induce their suppressive activity under acidic pH conditions [108]. Probiotics may also inhibit the secretion of bacterial toxins by the production of organic acids and because they have certain binding properties. For instance, in vitro bacterial co-incubation experiments and studies in mice have shown that different strains of *Bifidobacterium* were able to inhibit the expression of shiga toxin produced by *Escherichia coli* O157:H7, possibly due to the production of high amounts of organic acids such as acetic acid [109,110,111]. In addition, *Lactobacillus rhamnosus* strain GG has been able to bind mycotoxins, decreasing their bioavailability and potential interaction with intestinal cells in vitro [112]. Another antimicrobial activity of probiotic bacteria originates from bile acid de-conjugation. Bile acids have been documented to exhibit strong antimicrobial activity against different pathogens, like *Staphylococcus aureus* or *Salmonella typhimurium* in vitro [113]. Furthermore, an antifungal activity of probiotic bacteria has been reported. In case of *Lactobacillus plantarum* IMAU10014 the production of 3-phenyllactic acid as well as benzeneacetic acid has been demonstrated to mediate a broad spectrum antifungal activity in vitro [114].

Probiotics are also able to mediate their protective effects through an improvement of the intestinal barrier and immunomodulatory effects [82]. The epithelial cell layer of the gut is a major part of the physical barrier and has the important task of maintaining epithelial integrity, but this presents a dilemma: on the one hand the invasion of pathogens has to be prevented, on the other hand nutrients need to be absorbed. This problem is solved by tight junctions controlling the paracellular transport of various molecules and supporting the barrier function. For several probiotic strains a regulative effect on tight junction proteins has been shown which in consequence resulted in an improved defense against pathogens. A treatment with *Escherichia coli Nissle* 1917 and some *Lactobacillus* strains caused an up-regulation of the tight junction proteins zonula occludens and occludin which has been observed in vitro and in vivo in mice [115,116,117,118]. In addition, secretory components, such as mucins, secretory IgA and AMPs play an important role in the maintenance of the intestinal barrier. Several bacteria and bacterial fermentation products are able to modulate the expression of mucins thereby affecting composition and thickness of the mucin layer as well as their anti-adhesive effects which has been demonstrated in vitro [97,98,119,120]. A direct contact of bacterial components with cellular surface molecules may also activate signaling cascades mediating further antimicrobial effects. Host cells are able to recognize bacterial structures by PRRs like TLRs or nucleotide-binding oligomerization (NOD)-like receptors (NLRs) resulting in the activation of intracellular signaling cascades inducing various antimicrobial immune reactions. Some lactic acid bacteria stimulate the secretory IgA production presumably mediated through an activation of DCs via TLR signaling which has been shown in both, in DCs isolated from human blood in vitro and in human intervention studies in vivo [121,122]. IgA, produced by plasma cells and secreted into the luminal space, interacts with pathogens and toxins, thereby preventing their epithelial interaction and invasion [123]. TLRs may also be involved in the probiotic modulation of the intestinal epithelial synthesis of antimicrobial peptides. For instance, the induction of β-defensin secretion has been shown for probiotic *Escherichia coli* and *Lactobacillus* in cell culture studies and in humans, respectively. However, the extent of modulation differs between probiotic bacterial strains [124,125,126].

Probiotics have also been shown to mediate immunomodulatory effects by affecting cytokine and chemokine production in cultured cells and in mice. For *Lactobacillus johnsonii* N6.2 an up-regulation of the expression of the chemokines CCL20, CXCL8, and CXCL10 in cultured intestinal epithelial cells being associated with an increased expression of TLR7 and 9 has been demonstrated [127]. *Bifidobacterium breve* has been shown to induce the IL-10 producing type 1 regulatory cells in a TLR2 dependent pathway in mice. However, the underlying pathway is still unknown. [128].

Some effects of probiotics resulting from cellular signaling cascades may be independent of a direct interaction with live bacteria. Since a direct interaction of bacterial members of the intestinal microbiota with epithelial cells has been rarely demonstrated, dissolved components of bacterial origin like DNA motifs, cell wall components or secreted substances may be mediating the observed probiotic effects [129,130]. Therefore, also isolated bacterial secretion products or inactivated bacteria may exhibit beneficial effects and may be considered for the use in immunocompromised people due to concerns of possible adverse effects of live bacteria [131]. Nevertheless, it has to be taken into account that opposite effects of inactivated bacteria may occur potentially resulting in adverse health effects [132,133].

#### 2.5.2. Prebiotics

The term prebiotic is defined as “a substrate that is selectively utilized by host microorganisms conferring a health benefit” [10]. Prebiotics include carbohydrate-based structures but also other compounds like polyphenols, which affect a limited range of microorganisms. Fructans and galactans being metabolized by *Bifidobacteria* are among the most frequently studied prebiotics [134]. Prebiotics are metabolized by various bacterial species including *Bifidobacteria* and *Bacteroides* that efficiently metabolize low-molecular weight carbohydrates and high molecular weight polysaccharides, respectively. However, bacterial degradation processes cannot be considered individually as ecological networks between bacteria exist consequently leading to processes of cross-feeding where metabolites of one microorganism are processed by another one resulting in different degradation products. Interestingly, oligofructose treatment resulted in an increased turnover of lactate- and acetate-producing bacteria resulting in high levels of butyrate in human fecal samples [135].

Several protective effects of prebiotics have been associated with their impact on the intestinal microbiota and the resulting metabolites. For example, the application of inulin or fructans has increased the number of *Bifidobacteria* and total SCFAs and decreased the pH in human feces [136,137]. Several bacterial metabolites have been associated with beneficial effects on host health. Especially SCFAs, organic acids and the amino acid tryptophan have been reported to exhibit positive effects on the control of pathogenic microorganisms, nutrient absorbance, immune homeostasis, maintenance of the intestinal epithelial barrier and impact on the gut-brain axis [138]. Various in vitro and in vivo studies have demonstrated important immune modulating properties as well as an improvement of the intestinal barrier following butyrate exposure. Besides its function as an energy source for colonocytes, direct anti-inflammatory effects of butyrate have also been observed, including the suppression of NFκB activation in the human intestinal cell line HT-29 [139]. In a Caco-2 cell culture model butyrate has been reported to enhance the intestinal barrier by affecting the assembly of tight junctions [84]. It has been shown that acetate and propionate producing *Bacteroides thetaiotaomicron* promote the differentiation of goblet cells and induce the expression of genes related to mucin production in the colonic epithelium of gnotobiotic rats [140].

The effects of prebiotics have been demonstrated to be highly dependent on the microbial ecosystem present and therefore may differ between different hosts [11]. In addition, the type, dosage and the concentration of prebiotics as well as the duration of intake are crucial for their health-promoting effects [141,142]. However, compounds that have been classified as prebiotics cannot be assessed for their individual effects on microorganisms. Potential direct effects cannot be analyzed as generated metabolites may be used by other bacterial members. Interestingly, it has been suggested that inulin and oligofructose meditate their direct effects through carbohydrate receptors located on intestinal epithelial and immune cells [143].

## 3. Conclusions

In conclusion, protective effects with regard to immune function and specifically towards inflammatory processes of various plant bioactives, including flavonoids, phenolic acids, stilbens and glucosinolates, and their break down products, have been documented in various in vitro and in vivo studies pointing towards a potential use as a drug or a nutritional supplement in the treatment of chronic inflammatory processes in the gut. Although initial clinical trials have been performed supporting the anti-inflammatory effects, additional studies are needed prior to recommending these plant bioactives for preventive and therapeutic use in patients with chronic inflammatory diseases.

Additionally, various effects of probiotics and prebiotics on the mucosal and the systemic immune system have been demonstrated. They have been shown to activate multiple mechanisms of the immune system and to modulate the residing microbiota offering a promising potential in the prevention or treatment of distinct inflammatory diseases including inflammatory bowel disease. However, it is important to keep in mind that different microbial species and prebiotic substances may mediate various effects and a continuous intake of probiotics and prebiotics may be necessary to ensure the described health-promoting effects.

## Figures and Tables

**Table 1:** Summary of various effects of selected plant bioactives on inflammatory markers in different model systems.

Compound	Model System	Inflammatory Markers	Reference
**Flavonoids**			
- Anthocyanins	Co-culture cell model	NFκB pathway ↓	[18,19]
		Pro-inflammatory cytokines ↓	
		Pro-inflammatory cytokines ↓	
	Mice	Chemokine (CCL2) ↑	[20]
**Phenolic acids**			
- Hydroxybenzoid acids			
o Vanillic acid	Murine peritoneal macrophages	TNFα ↓, IL-6 ↓, COX-2 ↓, PGE2 ↓, NO ↓	[25]
o Gallic acid	RAW 264.7 cells	NFκB ↓	[26]
	Mice	NFκB ↓, IL-6 ↓, pSTAT3Y6 ↑	[27]
	Murine allograft model	Treg ↑, T-cell activation ↓, T-cell number ↓	[28]
- Hydroxycinnamic acids			
o Ferulic acid	THP1 cells	IL-1β ↓, IL-6 ↓	[29]
	Rats	IL-1β ↓, IL-4 ↓, IL-6 ↓	[30]
o Curcumin	Murine BDMC	Treg ↑	[33]
	Caco-2 cells (co-culture)	TNFα ↓, IL-6 ↓	[34]
	Mice (IL-10−/−)	IFNγ ↔	[35]
**Stilbens**			
- Resveratrol	Caco-2 cells	NFκB ↓	[41,42]
	SW480 cells	TLR4 ↑, iNOS ↑	[42]
	Caco-2 cells	NFκB ↑	[44]
	Mice	SAA ↓, SIRT1 ↑	[45]
		Pro-inflammatory cytokines ↓, Anti-inflammatory cytokines ↑, Treg/Th17 ↑	[46,47,48]
	Rats	PGE1 ↓, COX-2 ↓, iNOS ↓	[48]
		PGE1 ↓, COX-2 ↓	[49]
**Glucosinolates/Isothiocyanates**			
- Sulforaphane	Mice	Pro-inflammatory cytokines ↓	[61]
	Colitic mice	Weight loss ↓, pro-inflammatory cytokines ↓, monocyte infiltration ↓, miRNA-155 ↓	[62,63]
- Allyl-Isothiocyanate	Colitic mice	infiltration of immune cells ↓	[66,67]
	Caco-2-, HT-29-, SW480 cells	HBD-2 ↑	[68,69]
- Di-indolylmethane/Indol-3-carbinol	Murine macrophages	Inflammatory markers ↓	[70]
	Jurkat cells	Inflammatory processes ↑ (high concentrations)	[75]

Abbreviations: BMDC—bone marrow derived dendritic cells; COX—Cyclooxygenase; HBD-2—human β-defensin 1; IFNγ—Interferon γ; IL—Interleukin; iNOS—inducible Nitric Oxide Synthase; miRNA-155—microRNA-155; NFκB—Nuclear Factor κB; PGE1/2—Prostaglandin E1/2; SAA—serum amyloid A; SIRT1—sirtuin 1; TLR4—Toll-like receptor 4; Treg—regulatory T-cells; TNFα—Tumor Necrosis Factor α; ↑—up-regulation; ↓—down-regulation, ↔ —unchanged.
